# Synthesis of Mixed Chitin Esters via Acylation of Chitin in Deep Eutectic Solvents

**DOI:** 10.3390/molecules28248132

**Published:** 2023-12-16

**Authors:** Yusuke Egi, Jun-ichi Kadokawa

**Affiliations:** Graduate School of Science and Engineering, Kagoshima University, 1-21-40 Korimoto, Kagoshima 890-0065, Japan

**Keywords:** acylation, acyl chloride, chitin, deep eutectic solvent, mixed ester derivative

## Abstract

The development of efficient derivatization methods of chitin, such as acylation, has been identified to confer new properties and functions to chitin. In this study, we investigate the synthesis of mixed chitin esters via the acylation of chitin in deep eutectic solvents (DESs) comprising 1-allyl-3-methylimidazolum chloride and 1,1,3,3-tetramethylguanidine based on a previous study that reported the development of efficient acylation of chitin in the DES to obtain single chitin esters. A stearoyl group was selected as the first substituent, which was combined with several bulky acyl and long oleoyl groups as the second substituents. After dissolution of chitin in the DES (2 wt%), the acylation reactions were conducted using stearoyl and the desired acyl chlorides for 1 h + 24 h at 100 °C in the resulting solutions. The IR and ^1^H NMR spectra of the isolated products confirmed the structures of mixed chitin esters with two different substituents. The substituent ratios in the derivatives, which were estimated via the ^1^H NMR analysis, were changed according to the feed ratios of two acyl chlorides.

## 1. Introduction

Polysaccharides are widely distributed in nature and show viral and specific biological roles [1,2]. Chitin and cellulose are the two representative structural polysaccharides, mainly present in the exoskeletons of crustacean shells and the cell walls of plants, respectively [3,4]. They comprise *N*-acetyl-d-glucosamine and d-glucose repeating units, respectively, linked through the same β(1→4)-glycosidic bonds. Because of the extended fibrous and stiff polysaccharide chain packing formed through a number of hydrogen bonds, however, these structural polysaccharides exhibit poor processability and feasibility for application as soft materials, such as thermoplastics. For such an application of natural polysaccharides, including cellulose, practically, the derivatization of hydroxy groups, e.g., acylation (ester derivatization), has widely been used on the various polysaccharide chains [5]. Cellulose acylates (ester derivatives) have been most extensively studied, in which some derivatives are practically used as thermoplastics [6,7]. For example, cellulose triacetate is the most well-known ester derivative, which is practically applied as a photographic film, an optical compensation film for liquid crystal display, and a protective film for polarizing plate [8]. Although cellulose triacetate is a thermoplastic, it is high-melting and thus nearly not melt-processable. The substituted groups of cellulose ester derivatives are known to exert a significant impact on their melt processability and other properties. For example, mixed cellulose esters with different substituents exhibit elevated molecular mobility owing to more free volume. Representatively, cellulose acetate butyrate presents better melt-processability and superior thermoplasticity to cellulose triacetate, attributed to a lower viscous flow temperature [9].

Compared with such cellulose ester derivatives, chitin acylates (ester derivatives) have not been greatly employed in practical applications although chitin is well-known as the second abundant polysaccharide after cellulose. Difficulty in the efficient utilization of chitin ester derivatives is essentially due to limited attempts at the development of the useful acylation methods for chitin from native sources to be substantially applied in practical fields [10,11]. Alongside a chitin acetate, which is one of the most extensively studied ester derivatives [12,13], some studies on the preparation of chitin ester derivatives with several substituents have been conducted under strong acidic conditions using perchloric and methanesulfonic acid media [14,15,16,17,18,19,20]. Due to such acidic conditions, however, a decrease in the molecular weight in the chitin main-chains frequently happens during the reaction. The acylation of chitin with a high degree of substitution (DSs) in homogeneous solutions has been achieved in the *N,N*-dimethylacetamide (DMAc)-LiCl solvent system [21,22]. In the subsequent study, the systematic investigation on the synthesis of chitin ester derivatives with different acyl substituents was demonstrated using alkanoic acids in the presence of *p*-toluenesulfonyl chloride and pyridine in a DMAc-LiCl system [23]. However, long dissolution reaction times (5 days and 100 h, respectively) were required in order to obtain the planned derivatives in this system. We have achieved the development of an efficient acylation of chitin using acylation reagents in an ionic liquid, 1-allyl-3-methylimidazolium bromide (AMIMBr) to produce chitin ester derivatives with the quite-high DSs [24] based on the fact that AMIMBr dissolves chitin in concentrations up to 4.8 wt% via simple operation [25]. Such acylation of chitin in AMIMBr was demonstrated using various acyl chlorides and coexisting *N,N*-dimethyl-4-aminopyridine (DMAP)/pyridine as catalyst and base, respectively, to produce the corresponding chitin ester derivatives with DSs = ~2. In the subsequent study, the acylation approach of chitin in AMIMBr was applied to obtain mixed chitin esters comprising two kinds of acyl substituents with the high total DSs, that is, a stearate group, combined with adamantoyl, cinnamoyl, 1-naphthoyl, and oleoyl substituents [26].

**Figure 1:**
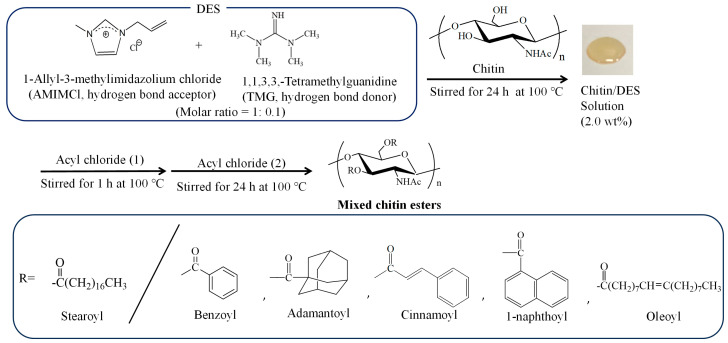
Synthesis of mixed chitin esters with stearoyl and different bulky and long acyl substituents in deep eutectic solvent (DES) composed of 1-allyl-3-methylimidazolium chloride (AMIMCl) and 1,1,3,3-tetramethylguanidine (TMG).

In the past decade, some deep eutectic solvents (DESs), as analogous solvents of ionic liquids, which mostly consist of choline chloride, have also been found for the dissolution of chitin, [27,28,29,30,31,32,33,34,35,36]. DESs are eutectic fluids which can be formed from favorable mixtures of hydrogen-bond acceptors and donors, and they show lower melting points than those of the individual hydrogen-bond acceptors/donors by self-associating through hydrogen bonding interactions [37]. We have also reported that DESs, which are composed of several methylimidazolium ionic liquids (as hydrogen-bond acceptors) and thiourea (as a hydrogen-bond donor), exhibit the ability to dissolve chitin in concentrations up to 5 wt% [38]. Based on the above background, we also investigated the acylation reaction of chitin in the presence of acyl chlorides in DESs comprising AMIMCl and several hydrogen-bond donors. DSs of the produced derivatives were significantly affected by the kinds of hydrogen-bond donors owing to their different basicity and nucleophilicity when acylation, such as hexanoylation of chitin, was representatively demonstrated using hexanoyl chloride in the presence of DMAP/pyridine in the DESs. Particularly, acylation of chitin was found to efficiently occur in the absence of DMAP/pyridine in a DES consisting of AMIMCl/1,1,3,3-tetramethylguanidine (TMG) owing to the high basicity and low nucleophilicity of TMG, which produces chitin ester derivatives with high DSs [39]. We also found that thermoplasticity was conferred to chitin benzoate stearates with appropriate substituent ratios, which were synthesized in AMIMBr, as mentioned above [40]. In the present study, based on the above backgrounds, we attempt to synthesize mixed chitin esters with long fatty and bulky acyl groups by means of the above acylation method in the DES composed of AMIMCl/TMG (Figure 1). The reaction took place smoothly with different combinations of acyl chlorides to obtain the corresponding mixed chitin eaters with different substituent ratios.

## 2. Results and Discussion

Prior to acylation, a 2 wt% chitin/DES (AMIMCl/TMG) solution was prepared according to the previously reported procedure [39]. A mixture of AMIMCl with TMG (molar ratio = 1:0.1) was first heated for 30 min at 100 °C to obtain the AMIMCl/TMG-DES. Chitin powder (2 wt%) was subsequently added to the DES and the resulting mixture was heated for 24 h at 100 °C for dissolution. Synthesis of mixed chitin esters in the obtained solution was conducted via the same acylation method as that used for single chitin esters in the DES.

We selected benzoyl and stearoyl groups as the substituents to initially investigate the acylation of chitin for the synthesis of mixed chitin esters because such derivatives with varying substituent ratios were precisely characterized after their synthesis in AMIMBr [40]. Therefore, chitin benzoate stearates were synthesized using benzoyl and stearoyl chlorides according to the acylation method in the AMIMCl/TMG-DES as follows (Figure 1). A mixture of benzoyl chloride with a 2 wt% chitin/DES solution was heated for 1 h at 100 °C. After stearoyl chloride was added, the resulting mixture was further heated for 24 h at 100 °C. A total amount of two acyl chlorides was adjusted to 20 equiv. with a repeating unit of chitin, where their several feed ratios were employed as listed in Table 1. The products were isolated as ethanol-insoluble fractions, which were characterized by IR and ^1^H NMR measurements. 

**Figure 2:**
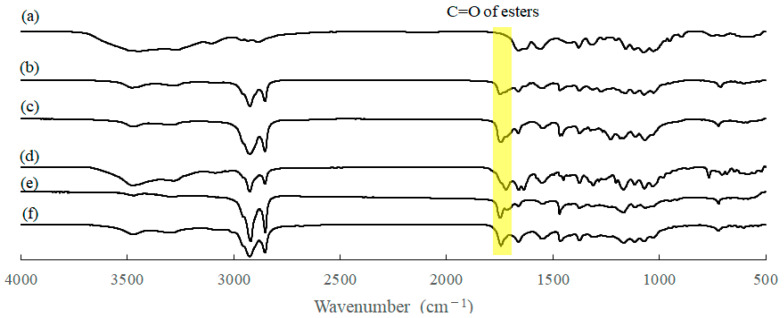
IR spectra of (**a**) chitin powder and (**b**–**f**) mixed chitin esters of entries 1, 4, 6, 8, and 10 in Table 1.

**Figure 3:**
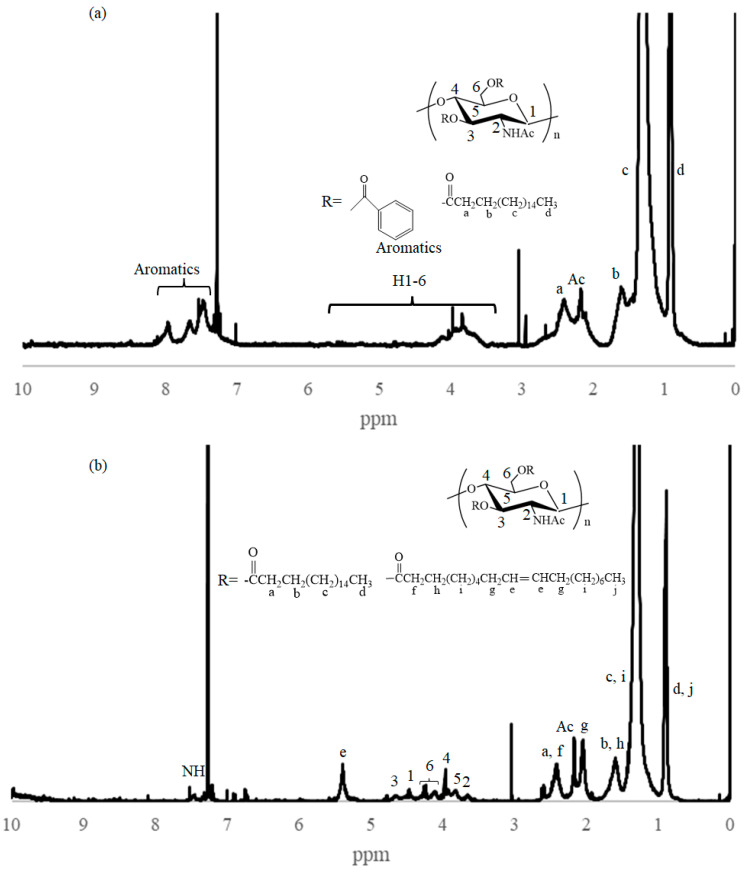
^1^H NMR spectrum of (**a**) chitin benzoate stearate (entry 1, Table 1) and (**b**) chitin oleate stearate (entry 10, Table 1) in CDCl_3_/CF_3_CO_2_H (2/1 in volume).

The IR spectrum of the product, obtained by a feed ratio of benzoyl/stearoyl chlorides = 9:1 (entry 1, Table 1), observed two new carbonyl absorptions at 1715 and 1740 cm^−1^ ascribed to aromatic and aliphatic esters, respectively, compared to that of chitin powder (Figure 2a,b). The spectroscopic pattern was the same as that of the chitin benzoate stearate prepared in AMIMBr in our previous study [40]. The IR result indicated the progress of acylation under the above conditions in the DES to produce the chitin benzoate stearate. The ^1^H NMR spectrum of the product in CDCl_3_/CF_3_CO_2_H (2/1 in volume) also supported its structure because of the detection of both benzoyl and stearoyl signals, as shown in Figure 3a. However, the signals corresponding to H1–H6 in the *N*-acetyl-d-glucosamine repeating units were not clearly detected; this is owing to the shielding of the chitin chain by bulky benzoyl groups, as discussed in our previous study [40]. As the DS value of acyl groups was not exactly calculated due to this reason, the substitution ratio (SR) was alternatively estimated from the integrated ratio of the methyl signals of the stearoyl group to the aromatic signals of the benzoyl group (1.17:0.79), which was 1:0.41. As shown in Table 1 (entries 1–3, Table 1), the SR values were changed according to the feed ratios of two acyl chlorides. 

In the following investigation, stearoyl chloride was combined with the other bulky acyl chlorides, such as adamantoyl, cinnamoyl, and 1-naphthoyl chlorides, as well as unsaturated long fatty acyl chloride, that is, oleoyl chloride, in the acylation approach via the same operation in the DES to obtain the corresponding mixed chitin esters. The IR spectra of all the products using stearoyl and bulky acyl chlorides (entries 4–9, Table 1) newly exhibited carbonyl absorptions derived from ester linkages at around 1715–1747 cm^−1^, suggesting the progress of acylation (Figure 2c–e). The ^1^H NMR spectra of the products in CDCl_3_/CF_3_CO_2_H (2/1 in volume) observed the signals assignable to both groups to support the structures of the corresponding mixed chitin esters (Figures S1–S3). The detailed signal assignments are described in Section 3.2. Like the ^1^H NMR spectrum of chitin benzoate stearate (Figure 2a), the signals derived from the chitin main-chains were not clearly detected due to shielding by the bulky groups, leading to difficulty in calculating the exact DS values. Alternatively, the SR values were estimated from the integrated ratios of the methyl signals of the stearoyl groups to the signals ascribed to the bulky groups (methylene (-CH-C*H*_2_-CH-) signals for adamantoyl, methine (=CH-C=O) signals for cinnamoyl, and aromatic signals for 1-naphthoyl), which were changed according to the feed ratios of acyl chlorides. The ratios of the bulky acyl/stearoyl groups were always lower than the feed ratios of bulky acyl/stearoyl chlorides, indicating lower reactivity of the bulky acyl chlorides than that of stearoyl chloride in the present acylation system.

Different from the above ^1^H NMR results of the products having the bulky acyl substituents, the ^1^H NMR spectrum of chitin oleate stearate, prepared using oleoyl and stearoyl chlorides (entry 10, Table 1), showed the seven individual signals ascribable to the H1–H6 protons in *N*-acetyl-d-glucosamine units besides the signals derived from the substituted oleoyl and stearoyl groups because of the absence of bulky groups (Figure 3b). From the integrated ratios among the methyl signal, the alkenic methine signal, and the sugar signals (0.87:0.20:1), accordingly, the DS value of stearoyl and oleoyl groups were calculated to be 1.33 and 0.70, respectively (total 2.03). The ^1^H NMR result supported the production of chitin oleate stearate with the high DS value. The IR spectrum of the product exhibited a carbonyl absorption at 1740 cm^−1^ ascribed to aliphatic esters, also supporting the chitin oleate stearate structure (Figure 2f).

As the mechanism for the present efficient acylation process, the occurrence of halogen exchange between acyl chloride and AMIMBr is considered, which was already proposed in our previous study [26]. Acyl bromides are potentially produced in situ by such an exchange reaction in the systems. Then, the resulting acyl bromides with higher reactivity than acyl chlorides efficiently react with hydroxy groups in chitin to yield the mixed chitin esters with high SR values.

## 3. Materials and Methods

### 3.1. Materials

The crab shell chitin powder was purchased from FUJIFILM Wako Pure Chemical Corporation (Osaka, Japan). The ionic liquid, AMIMCl, was synthesized via quaternization of 1-methylimidazole (Tokyo Chemical Industry Co., Ltd., Tokyo, Japan) with 3-chloro-1-propene (FUJIFILM Wako Pure Chemical Corporation, Osaka, Japan) according to the method adapted from the literature procedure [41]. Other solvents and reagents were commercially available and used without further purification.

### 3.2. Synthesis of Mixed Chitin Esters with Stearoyl and Bulky Acyl Groups in DESs

A typical experimental procedure for synthesis of mixed chitin ester was as follows (entry 1, Table 1): An AMIMCl (2.0 g, 12.6 mmol)/TMG (0.145 g, 1.26 mmol) mixture was heated for 30 min at 100 °C to produce the AMIMCl/TMG-DES. After chitin powder (0.0420 g, 0.207 mmol) was added to the prepared DES, the mixture was stirred for 24 h at 100 °C to give a 2.0 wt% solution. Benzoyl chloride (0.414 mL, 3.6 mmol, 18 equiv. with a repeating unit) was then mixed into the solution, and the mixture was heated for 1 h at 100 °C with stirring. After stearoyl chloride (0.136 mL, 0.4 mmol, 2 equiv. with a repeating unit) was additionally mixed, the resulting mixture was further heated for 24 h at 100 °C with stirring. Ethanol (200 mL) was then added to the reaction mixture to precipitate the product. The precipitated product was isolated by filtration, which was washed with ethanol and dried under reduced pressure for 1 h at 60 °C to yield the chitin benzoate stearate (0.136 g). IR: 1715, 1740 cm^−1^ (C=O of ester); ^1^H NMR (Figure 3a, CDCl_3_/CF_3_CO_2_H (2/1 in volume)) δ 0.92 (br t, *J* = 6.4 Hz, C*H*_3_CH_2_-), 1.33 (br s, CH_3_(C*H*_2_)_14_-), 1.62 (br s, -C*H*_2_CH_2_C=O), 2.16 (br s, CH_3_C=O), 2.41 (br s, -CH_2_C=O), 3.52–5.65 (m, H1–H6), and 7.32–8.24 (m, aromatics, NH). The SR values were calculated from integrated ratios of methyl signals of stearoyl groups to aromatic signals of benzoyl groups (1.17:0.79, 1.87:0.33, and 2.45:0.30 for entries 1–3).

The other mixed chitin esters with stearoyl and adamantoyl, cinnamoyl, or 1-naphthoyl groups were synthesized via the same procedure as above using the corresponding acyl chlorides.

Chitin adamantate stearate: IR 1740 cm^−1^ (C=O of ester), ^1^H NMR (Figure S1, CDCl_3_/CF_3_CO_2_H (2/1 in volume)) δ 0.92 (br t, *J* = 6.4 Hz, C*H*_3_CH_2_-), 1.34 (br s, CH_3_(C*H*_2_)_14_-), 1.62 (m, -C*H*_2_CH_2_C=O), 1.82 (m, -CH-C*H*_2_-CH-), 1.93–2.11 (m, CH_3_C=O, -CH-, -CH_2_-C-C=O), 2.39 (br s, -CH_2_C=O), 3.52–5.84 (m, H1–H6), and 7.41 (br s, NH). The SR values were calculated from integrated ratios of methyl signals of stearoyl groups to methylene (-CH-C*H*_2_-CH-) signals of adamantoyl groups (1.38:0.59 and 2.07:0.08 for entries 4 and 5).

Chitin cinnamate stearate: IR 1720, 1747 cm^−1^ (C=O of ester), ^1^H NMR (Figure S2, CDCl_3_/CF_3_CO_2_H (2/1 in volume)) δ 0.92 (br t, *J* = 6.4 Hz, C*H*_3_CH_2_-), 1.31 (br s, CH_3_(C*H*_2_)_14_-), 1.52 (br s, -C*H*_2_CH_2_C=O), 2.13 (s, CH_3_C=O), 2.38 (br s, -CH_2_C=O), 3.21–5.57 (m, H1–H6), 6.48 (br s, =CH-C=O), 7.30–7.69 (br, aromatics, NH), and 7.75 (br s, =CH-Ar). The SR values were calculated from integrated ratios of methyl signals of stearoyl groups to methine (=CH-C=O) signals of cinnamoyl groups (0.75:0.09 and 2.09:0.02 for entries 6 and 7).

Chitin 1-naphthoate stearate: IR 1715, 1740 cm^−1^ (C=O of ester), ^1^H NMR (Figure S3, CDCl_3_/CF_3_CO_2_H (2/1 in volume)) δ 0.89 (br t, *J* = 6.0 Hz, C*H*_3_CH_2_-), 1.29 (br s, CH_3_(C*H*_2_)_14_-), 1.63 (br s, -C*H*_2_CH_2_C=O), 2.15 (br s, CH_3_C=O), 2.43 (br s, -CH_2_C=O), 3.35–5.34 (m, H1–H6), and 7.31–8.36 (m, aromatics, NH). The SR values were calculated from integrated ratios of methyl signals of stearoyl groups to aromatic signals of 1-naphthoyl groups (10.37:4.14 and 2.09:0.16 for entries 8 and 9).

### 3.3. Synthesis of Mixed Chitin Oleate Stearate in DES (Entry 10, Table 1)

An AMIMCl (2.0 g, 12.6 mmol)/TMG (0.145 g, 1.26 mmol) mixture was heated for 30 min at 100 °C to produce the AMIMCl/TMG-DES. After chitin powder (0.0420 g, 0.207 mmol) was added to the prepared DES, the mixture was stirred for 24 h at 100 °C to give a 2.0 wt% solution. Stearoyl chloride (0.680 mL, 2.0 mmol, 10 equiv. with a repeating unit) was then mixed to the solution, and the mixture was heated for 1 h at 100 °C while stirring. After oleoyl chloride (0.690 mL, 2.0 mmol, 10 equiv. with a repeating unit) was additionally mixed, the resulting mixture was further heated for 24 h at 100 °C with stirring. Ethanol (200 mL) was then added to the reaction mixture to precipitate the product. The precipitated product was isolated by filtration, which was washed with ethanol and dried under reduced pressure for 1 h at 60 °C to yield the chitin benzoate stearate (0.105 g). IR 1740 cm^−1^ (C=O of ester); ^1^H NMR (Figure 3b, CDCl_3_/CF_3_CO_2_H (2/1 in volume)) δ 0.89 (br t, *J* = 6.0 Hz, C*H*_3_CH_2_-), 1.27 (br s, -CH_3_(C*H*_2_)_14_-, -(C*H*_2_)*_6_*CH_2_CH=, -(C*H*_2_)_4_CH_2_CH=), 1.58 (br s, -C*H*_2_CH_2_C=O), 2.04 (br s, C*H*_2_CH=), 2.16 (br s, CH_3_C=O), 2.39 (br s, -CH_2_C=O), 3.55–4.79 (m, H1–H6), 5.47 (br s, -CH=), and 7.45 (br s, NH). The DS and SR values were calculated from integrated ratios among methyl signal, alkenic methine signal, and sugar signals (0.87:0.20:1).

### 3.4. Measurements

IR spectra were recorded on a PerkinElmer Spectrum Two spectrometer (PerkinElmer Japan Co., Ltd., Yokohama, Japan). The ^1^H NMR spectra were recorded using an ECX400 instrument (JEOL, Akishima, Tokyo, Japan).

## 4. Conclusions

In this paper, we reported the synthesis of mixed chitin esters with stearoyl and several bulky acyl and oleoyl groups via acylation in the AMIMCl/TMG-DESs. The reactions were conducted using stearoyl and the corresponding acyl chlorides for 1 h + 24 h at 100 °C in the 2 wt% chitin/DES solutions. The products were isolated as ethanol-insoluble fractions, which were characterized via the IR and ^1^H NMR measurements. The resulting spectra of all the products fully supported the corresponding mixed chitin ester structures. The substituent ratios in the derivatives were changed according to the feed ratios of the two acyl chlorides. We have already found, in a previous study, that chitin benzoate stearates with appropriate substituent ratios show thermoplasticity, associated with the regular parallel stearoyl packings [40]. Because we have developed the efficient acylation method of chitin in the DES in this study, we will investigate new properties and functions from the other mixed chitin esters with different acyl substituents, such as heterocyclic acyl groups, obtained via the present acylation method, and their potential to be practically applied as chitin-based materials in the future.

## Figures and Tables

**Table 1:** Synthesis of mixed chitin esters using stearoyl and second acryl chlorides in a deep eutectic solvent (DES) composed of 1-allyl-3-methylimidazolium chloride (AMIMCl) and 1,1,3,3-tetramethylguanidine (TMG) ^(a)^.

Entry	Stearoyl Chloride (Equiv.) ^(b)^	Second Acyl Chloride (Equiv.) ^(b)^	Yield (g)	Substituent Ratio (SR) ^(c)^ (Stearoyl:Second Acyl)
1	2	benzoyl (18)	0.136	1:0.41
2	10	benzoyl (10)	0.138	1:0.11
3	18	benzoyl (2)	0.163	1:0.08
4	10	adamantoyl (10)	0.102	1:0.16
5	18	adamantoyl (2)	0.157	1:0.01
6	10	cinnamoyl (10)	0.130	1:0.36
7	18	cinnamoyl (2)	0.156	1:0.03
8	10	1-naphthoyl (10)	0.141	1:0.17
9	18	1-naphthoyl (2)	0.117	1:0:03
10	10	oleoyl (10)	0.105	1:0.53 (DS (stearoyl) = 1.33, DS (oleoyl) = 0.70) ^(d)^

^(a)^ Reaction was conducted using chitin (0.0420 g, 0.207 mmol) with stearoyl and second acyl chlorides (total; 20 equiv. with a repeating unit of chitin) for 24 h at 100 °C in 2 wt% solution (molar ratio of AMIMCl/TMG = 1:0.1). ^(b)^ With a repeating unit. ^(c)^ Substituent ratio determined via ^1^H NMR measurement. ^(d)^ Degree of substitution determined via ^1^H NMR measurement.

## Data Availability

Data are contained within the article and Supplementary Materials.

## Supplementary Materials

The following supporting information can be downloaded at https://www.mdpi.com/article/10.3390/molecules28248132/s1: Figure S1: ^1^H NMR spectrum of chitin adamantate stearate (entry 4, Table 1) in CDCl_3_/CF_3_CO_2_H (2/1 in volume); Figure S2: ^1^H NMR spectrum of chitin cinnamate stearate (entry 6, Table 1) in CDCl_3_/CF_3_CO_2_H (2/1 in volume); Figure S3: ^1^H NMR spectrum of chitin 1-naphthoate stearate (entry 9, Table 1) in CDCl_3_/CF_3_CO_2_H (2/1 in volume).
