# Supplementary material for: Synthesis of Mixed Chitin Esters via Acylation of Chitin in Deep Eutectic Solvents
Source: Molecules. 2023 Dec 16;28(24):8132. doi: 10.3390/molecules28248132 (PMC10745496; doi:10.3390/molecules28248132)
Supplement: Supplementary file 1 [file molecules-28-08132-s001.zip › molecules-2771743-supplementary.pdf]

Supporting Information

Synthesis of Mixed Chitin Esters via Acylation of Chitin in Deep Eutectic Solvents

Yusuke Egi and Jun-ichi Kadokawa*

Graduate School of Science and Engineering, Kagoshima University, 1-21-40 Korimoto, Kagoshima 890-0065, Japan

* E-mail: kadokawa@eng.kagoshima-u.ac.jp ; Tel.: +81-99-285-7743

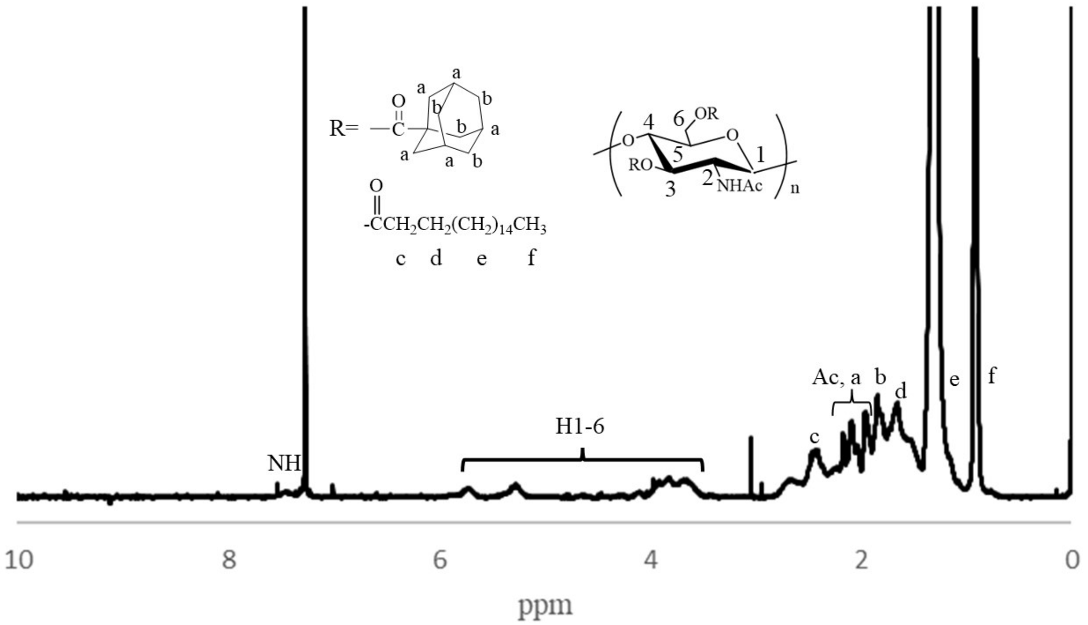

Figure S1 of chitin adamantate stearate (entry 4, Table 1) in $CDCl_3/CF_3CO_2H$ (2/1 in volume).

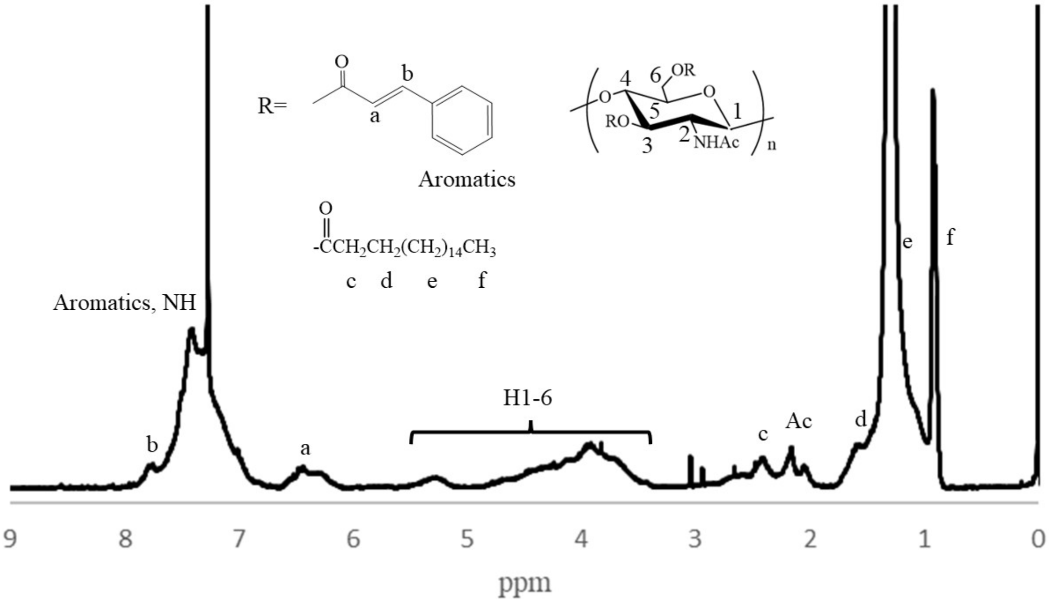

Figure S2 ^{1}H NMR spectrum of chitin cinnamate stearate (entry 6, Table 1) in $CDCl_3/CF_3CO_2H$ (2/1 in volume).

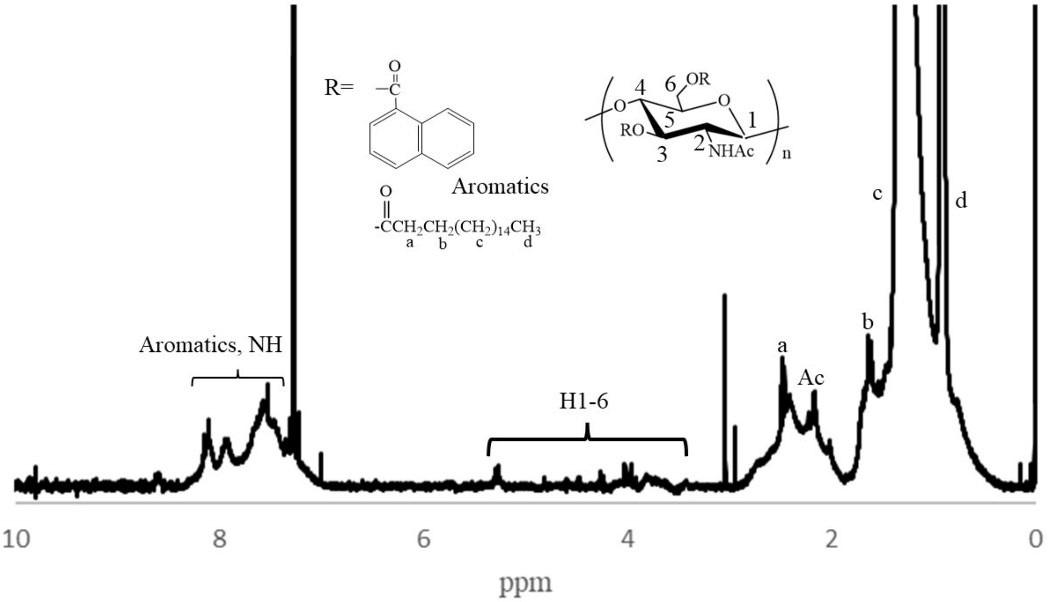

Figure S3 ^{1}H NMR spectrum of chitin 1-naphthoate stearate (entry 9, Table 1) in $CDCl_3/CF_3CO_2H$ (2/1 in volume).
